# Mitochondrial fission inhibitor Mdivi-1 alleviates lipopolysaccharide-induced parvalbumin interneurons dysregulation and cognitive impairments in a mouse model of sepsis-associated encephalopathy

**DOI:** 10.3389/fphar.2025.1525028

**Published:** 2025-06-12

**Authors:** Lei Dai, Shuxin Gu, Yibao Zhang, Siqi Ma, Peishan Wang, Jingyun Zhang, Cai Wang, Gaowei Su, Qun Fu, Wei Zhou, Yunxia Fan

**Affiliations:** ^1^ Department of Anesthesiology, Jintan Affiliated Hospital of Jiangsu University, Changzhou, China; ^2^ Jiangsu Key Laboratory of Medical Science and Laboratory Medicine, School of Medicine, Jiangsu University, Zhenjiang, Jiangsu, China; ^3^ Department of Anesthesiology, Jinling Hospital, Medical School of Nanjing University, Nanjing, China; ^4^ Department of Anesthesiology, Nanjing Drum Tower Hospital, Affiliated Hospital of Medical School, Nanjing University, Nanjing, China; ^5^ Department of Anesthesiology, Renji Hospital, School of Medicine, Shanghai Jiaotong University, Shanghai, China

**Keywords:** sepsis-associated encephalopathy, Mdivi-1, parvalbumin interneurons, neuroinflammation, cognitive impairment

## Abstract

**Introduction:**

Dysregulation of parvalbumin (PV) interneurons has been implicated in sepsis-associated encephalopathy (SAE), yet the underlying mechanisms remain poorly understood, and effective treatments are lacking. Given the high energy demands of PV interneurons and the emerging role of mitochondrial dynamics in SAE pathophysiology, this study aimed to investigate whether the mitochondrial fission inhibitor Mdivi-1 could alleviate PV interneuron dysfunction and cognitive impairments in a mouse model of SAE.

**Methods:**

C57BL/6 male mice were injected with lipopolysaccharide (LPS) to establish an animal model of SAE. Mdivi-1 was administered intraperitoneally 1 h before LPS challenge. Hippocampal tissues were harvested 24 h after LPS challenge for biochemical and histochemical analyses, and mitochondrial morphology was evaluated using transmission electron microscopy. In vivo electrophysiology and behavioral tests were performed between 2 and 4 days after LPS challenge to measure neural oscillations in the hippocampus and assess cognitive function.

**Results:**

Our results showed that LPS induced neuroinflammation, mitochondrial fission abnormalities, ATP depletion, and downregulation of PV interneurons in the hippocampus, collectively contributing to reduced gamma oscillations and cognitive impairments in mice. However, these effects were mitigated by Mdivi-1 treatment.

**Conclusion:**

Our study suggests that Mdivi-1 may offer a promising therapeutic approach for attenuating PV interneurons dysfunction and cognitive impairments in SAE.

## 1 Introduction

Sepsis-associated encephalopathy (SAE) is characterized by diffuse cerebral dysfunction without direct infection of the central nervous system. Survivors of severe sepsis may experience various cognitive impairments, including deficits in attention, executive function, memory, and information processing speed (Iwashyna et al., 2010). These cognitive deficits are often associated with dysfunction in the hippocampus (Gunther et al., 2012; Widmann and Heneka, 2014). The pathogenesis of SAE involves several factors, including blood-brain barrier (BBB) breakdown, neuroinflammation, glial activation, abnormal redox signaling, and mitochondrial structural and functional abnormalities. However, the cellular and molecular mechanisms of SAE remain unclear, resulting in a lack of effective pharmacological treatments (Wu et al., 2015).

Gamma oscillations (30–80 Hz) are high-frequency brain rhythms arising from the synchronized firing of neuronal populations, particularly fast-spiking GABAergic interneurons such as parvalbumin (PV)-expressing cells (Sohal, 2022; Inan et al., 2016; Milicevic et al., 2024). These oscillations are thought to facilitate the coordination of neural activity across different brain regions, enabling efficient information processing. Gamma activity has been closely associated with key cognitive and behavioral functions, including sensory representation, learning, working memory, emotional processing, selective attention and perception (Lundqvist et al., 2016; Deng et al., 2024). Disruption of gamma oscillations can impair these essential cognitive processes and has been implicated in various neurological and psychiatric conditions, including schizophrenia, Alzheimer’s disease, and epilepsy (Sohal, 2022; Guan et al., 2022).

Recent studies suggest that neuroinflammatory processes, such as those observed in SAE, may disrupt PV interneuron function, leading to impaired gamma synchronization and subsequent cognitive dysfunction (Ji et al., 2015; Ji et al., 2020). However, the mechanisms underlying PV interneuron dysfunction during SAE remain poorly understood. Lipopolysaccharide (LPS)-induced inflammation has been shown to selectively reduce PV expression in the medial prefrontal cortex (Ji et al., 2020). Additionally, systemic inflammation decreases hippocampal PV expression, leading to impairments in hippocampus-dependent learning and memory (Mao et al., 2021). Due to their fast-spiking nature and involvement in neural oscillations, PV interneurons have high energy demands, making them particularly vulnerable to energy deficits. Furthermore, hippocampal PV interneurons are reported to have higher mitochondrial content compared to other neurons (Gulyás et al., 2006). Thus, mitochondrial dysfunction may contribute to dysregulation of PV interneurons and abnormalities in neural oscillations and complex information processing deficits (Kann et al., 2014).

Mitochondria are highly dynamic organelles that continuously undergo fission and fusion to maintain their morphology and function. Dynamin-related protein 1 (DRP1) is a key mediator of mitochondrial fission, while OPA1 (optic atrophy 1) is essential for fusion (Schmitt et al., 2018; Yang et al., 2022). These dynamics are critical for meeting the energy demands of cells, particularly neurons, which rely heavily on efficient mitochondrial function for synaptic activity and survival (Morton et al., 2021). Previous studies have shown that sepsis can lead to excessive mitochondrial fission, contributing to energy deficits, cellular dysfunction, and neuronal damage (Fu et al., 2024; Deng et al., 2018). Mitochondrial division inhibitor-1 (Mdivi-1), a selective inhibitor of the fission protein DRP1, has been reported to alleviate BBB disruption and neuronal cell death in models of traumatic brain injury (Wu et al., 2016; Song et al., 2019) and LPS-induced brain damage (Deng et al., 2018). These protective effects are thought to be mediated through the preservation of mitochondrial morphology and the inhibition of neuronal apoptosis (Wu et al., 2018; Deng et al., 2018).

However, whether Mdivi-1 can protect against PV interneurons dysregulation and associated abnormalities in neural oscillations in the context of SAE remains unknown. This study aims to evaluate the impact of Mdivi-1 on mitochondrial dynamics, PV interneuron function, and cognitive outcomes in an LPS-induced mouse model of SAE.

## 2 Materials and methods

### 2.1 Animals

C57BL/6 male mice, aged 10 weeks, were obtained from the Animal Center of Jintan Affiliated Hospital of Jiangsu University, Changzhou, China. All procedures involving animals were conducted in accordance with the National Institutes of Health Guidelines for the Care and Use of Laboratory Animals and were approved by the Institutional Animal Care and Use Committee of Jiangsu University. The mice were housed in groups of four per cage under a 12-hour light/dark cycle (lights on at 08:00) at a temperature of 24°C ± 1°C and humidity of 50% ± 10%, with *ad libitum* access to water and food.

### 2.2 Experimental protocols

Lipopolysaccharide (LPS), a cell wall component of Gram-negative bacteria and a potent activator of the innate immune system, is commonly used to establish the animal model of SAE (Anderson et al., 2015) and to investigate cognitive deficits in neurodegenerative diseases (Perry et al., 2007). Intraperitoneal (*i.p*.) administration of LPS is cost-effective, easy to use, noninvasive, and can induce a long-term neuroinflammatory state along with impairments in learning and memory performance (Savi et al., 2021). Therefore, LPS-induced neuroinflammation was employed to a mouse model of SAE in this study.

After 7 days of acclimatization, mice were randomly assigned to one of four experimental groups: (1) control + vehicle (Con + vehicle); (2) control + Mdivi-1 (Con + Mdivi-1); (3) LPS + vehicle (LPS + vehicle); and (4) LPS + Mdivi-1 (LPS + Mdivi-1). To induce a severe sepsis phenotype, LPS (5 mg/kg, *E. coli* serotype 0111: B4, Sigma‒Aldrich, USA) was administered intraperitoneally (*i.p.*) (Savi et al., 2021; Gao et al., 2015; Qin et al., 2007). Mice in the control groups received an equivalent volume (0.2 mL) of normal saline. According to the group assignments, Mdivi-1 (10 mg/kg, *i. p.*, Topscience Co. Ltd, China) or vehicle (1% DMSO) was administered 1 h prior to the LPS injection (Yang et al., 2022; Rappold et al., 2014). All injections were performed at 8:00 and 10:00 a.m. The LPS dose (5 mg/kg) was selected based on established models demonstrating its ability to induce systemic inflammation, BBB disruption, neuroinflammation, and cognitive impairments in rodents, closely mimicking clinical features of SAE. This dose is widely accepted as a clinically relevant model of severe sepsis in experimental research (Wang et al., 2022; Guo et al., 2024). The Mdivi-1 dose (10 mg/kg, i.p.) was chosen based on prior reports demonstrating that this concentration effectively reduces DRP1-mediated mitochondrial fission, preserves mitochondrial integrity, and exerts neuroprotective effects in models of neuroinflammation and intracerebral hemorrhage-induced secondary brain injury (Yang et al., 2022; Zhang Y. et al., 2021). Notably, Mdivi-1 has been shown to cross the BBB and act within the central nervous system (Wu et al., 2018; Zhang Y. et al., 2021; Rappold et al., 2014).

**FIGURE 1:**
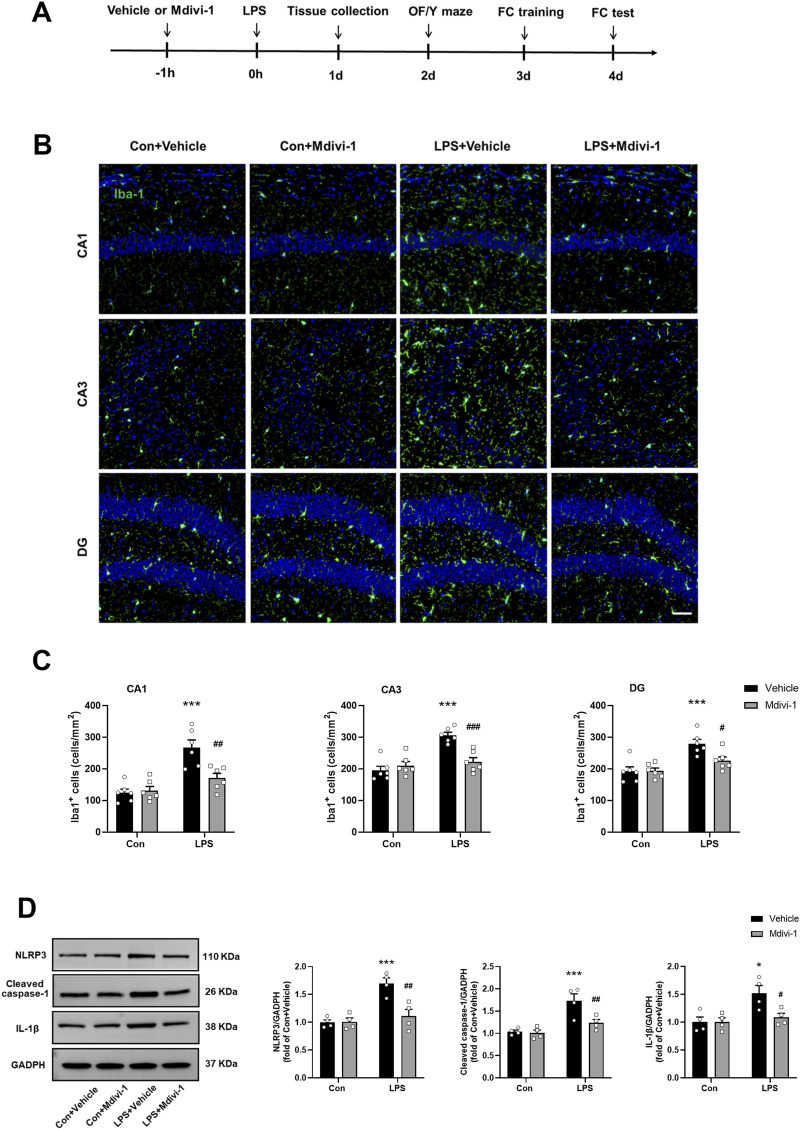
Mdivi-1 attenuated LPS-induced neuroinflammation in the mouse hippocampus. **(A)** Schematic timeline of the experimental procedure. **(B)** Quantification of the number of Iba-1 staining cells in the hippocampus among the four groups (n = 6 per group). **(C)** Representative immunofluorescence images showing Iba-1 (green) and DAPI (blue) staining in the hippocampus. Scale bar = 50 μm. **(D)** Representative Western blotting and quantitative analysis of NLRP3, cleaved caspase-1, and IL-1β protein levels in the hippocampus (n = 4 per group). Data are presented as the mean ± SEM. *P < 0.05, ***P < 0.001 compared to the Con + vehicle group, ^#^ *P* < 0.05, ^##^ *P* < 0.01, ^###^ *P <* 0.001 compared to the LPS + vehicle group.

Mice were anesthetized with 2% sodium pentobarbital (40 mg/kg, *i. p.*; Sigma, St. Louis, MO, USA) 24 h after LPS injection, and the brain and hippocampus were removed for biochemical and histochemical analyses and transmission electron microscopy. *In vivo* electrophysiology and behavioral tests were performed between 2 and 4 days after LPS challenge to measure neural oscillations in the hippocampus and assess cognitive function. The experimental protocol was detailed in Figures 1A, 5A.

### 2.3 Western blotting analysis

Hippocampi from each mouse were harvested and homogenized in ice-cold lysis buffer containing protease inhibitors, then centrifuged at 12,000 × g for 10 min at 4°C. The supernatant was collected, and protein concentration was measured using the Bradford assay. Twenty micrograms of protein were separated by SDS-PAGE and transferred to nitrocellulose membranes. After blocking with 5% nonfat dry milk for 1 h at room temperature, membranes were incubated overnight at 4°C with primary antibodies: DRP1 (1:1,000; Abbkine, China), OPA1 (1:1,000; Servicebio Technology Co. Ltd, China), nucleotide-binding domain-like receptor protein 3 (NLRP3, 1:500; Servicebio Technology Co. Ltd, China), cleaved caspase-1 (1:500; Servicebio Technology Co. Ltd, China), interleukin-1β (IL-1β, 1:500; Servicebio Technology Co. Ltd, China), glutamic acid decarboxylase 67 (GAD67, 1:1000; Servicebio Technology Co. Ltd, China), PV (1:1000; Servicebio Technology Co. Ltd, China), somatostatin (SST, 1:1000; Servicebio Technology Co. Ltd, China), GluR1 (1:1000; Servicebio Technology Co. Ltd, China) and GADPH (1:1000; Servicebio Technology Co. Ltd, China). Protein bands were visualized using enhanced chemiluminescence and quantified with ImageJ software (National Institutes of Health, Bethesda, MD, USA).

### 2.4 Adenosine triphosphate (ATP) content assays

Hippocampal tissues were harvested and homogenized in ice-cold lysis buffer supplemented with a protease inhibitor cocktail. The supernatant was collected by centrifugation at 12,000 × g for 5 min at 4°C. ATP content was measured with a firefly luciferase-based ATP assay kit (Beyotime Biotechnology, Shanghai, China). All measurements were performed according to the manufacturer’s instructions. Data were normalized to the control + vehicle group values and expressed as a percentage of control levels.

### 2.5 Transmission electron microscopy (TEM)

Hippocampi were removed and immediately put into Petri dishes with TEM fixative, and cut into 1 mm^3^ tissue blocks in the fixative, then transferred into 4°C for preservation and transportation. The tissues were postfixed with 1% osmium tetroxide in 0.1 M PBS (pH 7.4) for 2 h at room temperature. After that, the tissues were rinsed in 0.1 M PBS for 3 times, 15 min each. Tissues were then dehydrated through a graded series of ethanol, embedded in resin, and cut to 60–80 nm thin sections on an ultramicrotome, and the tissues were removed onto the 150 mesh copper grids with formvar film. A 2% uranium acetate saturated alcohol solution was used to avoid light staining for 8 min, rinsed in 70% ethanol 3 times and then rinsed in ultrapure water 3 times. Lead citrate (2.6%) was used to avoid CO2 staining for 8 min, and then the samples were rinsed with ultrapure water 3 times. After drying with filter paper, the cuprum grids were placed on a grid board and dried overnight at room temperature, then observed under TEM (HITACHI, HT7700, Japan). Three independent sections of the hippocampus were obtained and analyzed from each mouse, with three mice included in each group.

### 2.6 Immunofluorescence

Mice were anesthetized with 2% sodium pentobarbital and perfused intracardially with saline followed by 4% paraformaldehyde in phosphate-buffered saline (PBS). Brains were extracted, postfixed in 4% paraformaldehyde for 2 h, dehydrated in 30% sucrose at 4°C overnight, and embedded in optimal cutting temperature compound. Coronal brain sections with a thickness of 10 μm were prepared, with a 30 μm interval between each section. Sections were blocked with 1% bovine serum albumin (BSA) for 1 h at room temperature, and followed by primary antibodies: rabbit anti-ionized calcium-binding adaptor molecule-1 (Iba-1, 1:200, Abways, China), anti-GAD67 (1:200; ABclonal, China), anti-PV (1:200; ABclonal, China), anti-SST (1:200; ABclonal, China), and anti-DRP1 (1:200; Abbkine, China). Afterward, sections were incubated for 1 h with secondary antibodies: donkey anti-rabbit IgG-FITC (1:500, Abbkine Scientific Co., Ltd., Wuhan, China) or goat anti-rabbit IgG-Cy3 (1:600, Abbkine Scientific Co., Ltd., Wuhan, China) at room temperature. Fluorescence images were captured using a confocal microscope (Leica, TCS SP2, Germany). Co-expression of DRP1 with PV or SST was determined by counting neurons that were clearly and independently identifiable in both the red and green fluorescence channels, along with DAPI nuclear staining. Quantification was performed using ImageJ software (National Institutes of Health, Bethesda, MD, USA).

### 2.7 *In vivo* electrophysiology

Mice were anesthetized with 2% sodium pentobarbital, and body temperature was kept stable under an electric blanket. To expose the skull, the muscle, fascia, and periosteum were removed. After balancing the skull bone, three dimensional coordinates of the hippocampal CA1 area (DP, −2.0 mm; ML, ±1.5 mm; DV, −1.5 mm) were gained from the mouse brain atlas. A 3 × 3 mm window was drilled on the skull above the hippocampal CA1 area, and the cortex was fully exposed. An electrode array with 8 microwires was inserted via the window until the predetermined position was obtained. The window was sealed with paraffin, and the electrode was fixed with dental cement. After 7 days of recovery, local field potential (LFP) was recorded during the Y maze test on Day 2 after LPS challenge. To prevent interference, all other electric appliances were turned off during the recording session. The signals were transmitted into a digital preamplifier and digitized at a sampling rate of 40 kHz. Data processing was performed on a specific workstation and analyzed using NeuroExplorer 5 (Plexon Inc., Dallas, TX). The bands were filtered as follows: theta (4–7 Hz), alpha (8–13 Hz), beta (14–29 Hz), and gamma (30–80 Hz).

### 2.8 Open field (OF) test

The OF test was conducted on Day 2 after LPS challenge in a sound-isolated room. The test was performed in a white opaque plastic chamber (40 × 40 × 40 cm) to evaluate the exploratory and locomotor activities of the mice. Each mouse was gently placed in the center of the arena and allowed to explore for 5 min. The activities of the mice were automatically recorded with a video tracking system (XR-XZ301, XinRuan Information Technology Co., Ltd., Shanghai, China), and total ambulatory distance and time spent in the center were measured. To minimize any bias, a single investigator blinded to the treatment group and performed the behavioral tests. The equipment was cleaned with 75% ethanol between each experiment to eliminate any odor cues.

### 2.9 Y maze

The Y-maze test was performed 2 h after the OF test to assess spatial working memory in mice. It consisted of three arms at 120° angles that were labeled A, B, and C. Each animal was placed in the center of the Y maze and allowed to explore freely for 8 min throughout the three different arms of the equipment. The sequence and total number of entered arms were recorded. When the hind paws of mouse had been completely placed in the arm, arm entry was complete. For instance, a series of entries to the three arms ABC, ACBABACABA, would produce four “successful” alternations, ACB, CBA, BAC, and CAB. The score of alternation is the number of triads contained entries into all three arms divided by the maximum possible alternations (the total number of arms entered minus 2) × 100%. Re-entry into the same arm was not counted for analysis.

### 2.10 Fear conditioning (FC) test

To measure the associative memory, the fear conditioning test was conducted on Day 3 after LPS challenge using a chamber (50 cm high × 26 cm long × 26 cm wide). The test included two phases: training and testing. During the training phase, each animal was placed in a conditioning chamber and allowed to explore for 3 min, followed by 30-s tone (75 dB, 3 kHz) and a 3 s single electric foot shock (0.75 mA). Mice remained in the chamber for an additional 30 s before being returned to their cages. Twenty-four hours later, mice underwent the contextual fear conditioning test (a hippocampus-dependent task) by being reintroduced to the same chamber for 5 min without stimulation, and freezing behavior was recorded. Two hours after that, mice were placed in a novel chamber with different shape, color, and odor for the cued fear conditioning test (a hippocampus-independent task), which included a 3-min tone presentation. Cognitive deficits were assessed by measuring the amount of time the animal demonstrated “freezing behavior”, which is characteristic as a complete immobile posture except for respiratory efforts. Behavior activity was automatically recorded with a video tracking system (XR-XC404, XinRuan Information Technology Co., Ltd., Shanghai, China).

### 2.11 Statistical analysis

Statistical analyses were performed using GraphPad Prism 8.0 Software. Data are expressed as the mean ± SEM. These presented data were assessed for normal distribution by the Kolmogorov‒Smirnov test. Comparisons between two groups were performed by unpaired Student’s t*-*test. Differences among multiple groups were tested using two-factor ANOVA followed by Tukey’s multiple comparisons post-hoc test. A significant difference was considered as *P* < 0.05.

## 3 Results

### 3.1 Mdivi-1 attenuated LPS-induced neuroinflammation in the mouse hippocampus

To validate LPS-induced neuroinflammation, we first performed immunostaining with an antibody against Iba-1 (a marker of microglial activation) 24 h after LPS challenge. As shown in Figures 1B,C, compared to the Con + vehicle group, the number of Iba-1 staining cells were significantly increased in the hippocampal CA1 [*F* (1, 20) = 9.583, *P* = 0.0057], CA3 [*F* (1, 20) = 16.49, *P* = 0.0006] and DG [*F* (1, 20) = 4.974, *P* = 0.0374] in the LPS + vehicle group, indicating microglial activation. Notably, Mdivi-1 treatment reduced microglial activation in all examined hippocampal subregions following LPS challenge.

Our previous study indicated that sepsis activates the mitochondria-dependent NLRP3 inflammasome pathway and increases the release of inflammatory cytokines such as IL-1β (Zhang et al., 2023). Consistent with these findings, this study showed that the levels of NLRP3 [*F* (1, 12) = 14.25, *P* = 0.0026], cleaved caspase-1 [*F* (1, 12) = 5.801, *P* = 0.0330] and IL-1β [*F* (1, 12) = 5.065, *P* = 0.0440] were significantly elevated in the hippocampus of the LPS + vehicle group. However, these increases were not observed when Mdivi-1 was administered (Figure 1D).

### 3.2 Mdivi-1 rescued LPS-induced mitochondrial morphology alterations and restored ATP production in the mouse hippocampus

**FIGURE 2:**
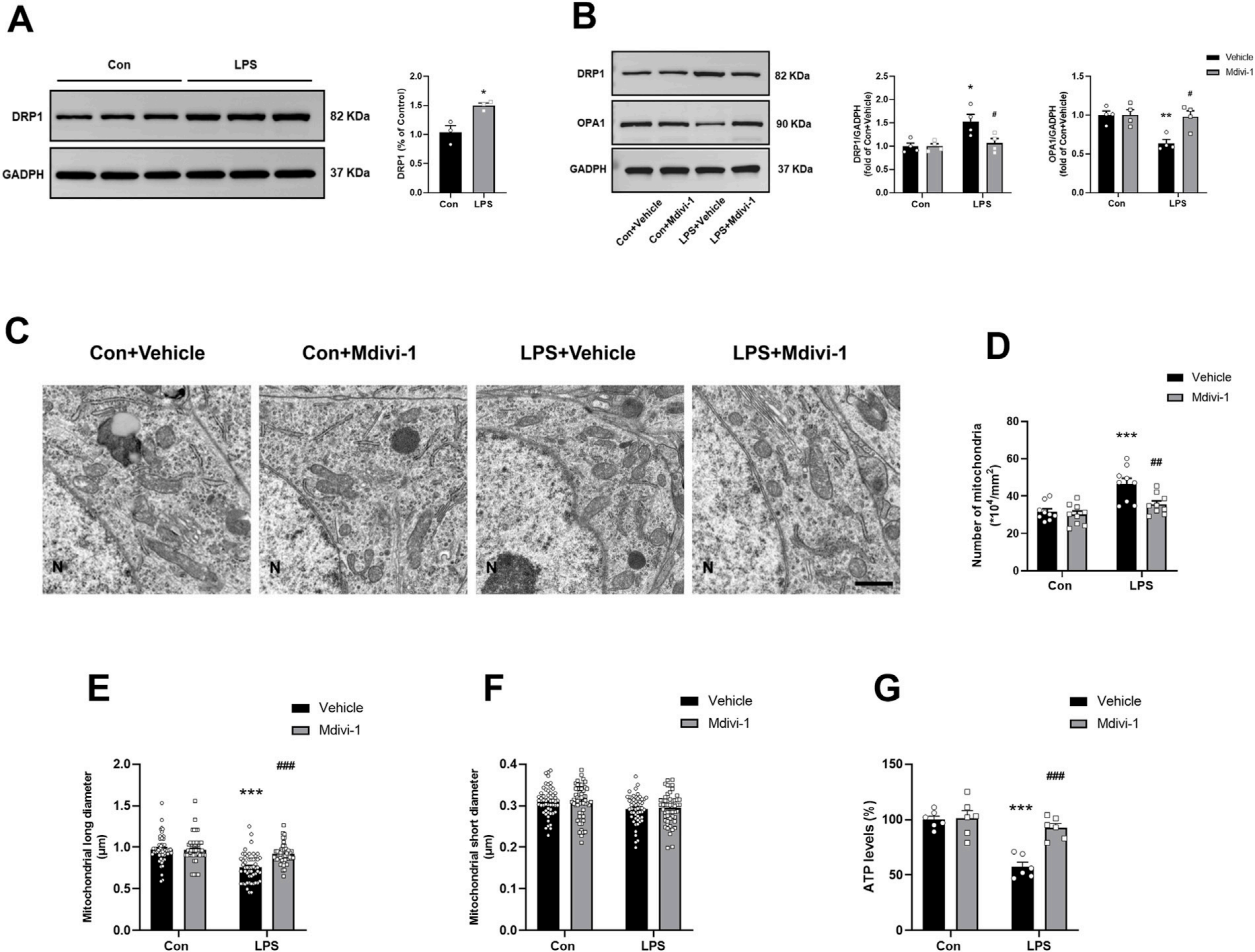
Mdivi-1 rescued LPS-induced mitochondrial morphology alterations and restored ATP production in the mouse hippocampus. **(A)** LPS induced an increase in the expression of DRP1 in the hippocampus (n = 3 per group). **(B)** Representative Western blotting and quantitative analysis of the protein levels of DRP1 and OPA1 in the hippocampus (n = 4 per group). **(C)** Representative TEM images showing mitochondrial ultrastructure in the hippocampus across the four groups. Magnification ×10,000. Scale bar = 1 µm. **(D)** Quantification of mitochondrial number in the hippocampus (n = 3 per group; three independent sections per mouse). **(E,F)** Quantification of mitochondrial long and short diameters in the hippocampus (n = 3 per group, three independent sections per mouse, 6 mitochondria randomly selected per section). **(G)** The ATP levels in the hippocampus (n = 6 per group). Data are presented as the mean ± SEM. *P < 0.05, **P < 0.01, ***P < 0.001 compared to the Con + vehicle group, ^#^ *P <* 0.05, ^##^ *P* < 0.01, ^###^ *P <* 0.001 compared to the LPS + vehicle group.

Mitochondrial morphology is regulated by a balance between fission and fusion proteins. DRP1 is a key mediator of mitochondrial fission, while OPA1 is essential for mitochondrial fusion (Schmitt et al., 2018; Yang et al., 2022). Previous studies have demonstrated that sepsis-induced neuroinflammation can promote excessive mitochondrial fission, contributing to neuronal injury and dysfunction. Consistent with these findings (Deng et al., 2018; Zhang et al., 2023), we observed that LPS treatment significantly increased DRP1 expression (*t* = 3.149, df = 4, *P* = 0.0345, Figure 2A) in the hippocampus of mice, indicating a shift toward excessive mitochondrial fission. Importantly, pretreatment with Mdivi-1 effectively reversed these LPS-induced changes, significantly reducing DRP1 expression and restoring OPA1 levels toward those observed in controls [*F* (1, 12) = 5.756, *P* = 0.0336; *F* (1, 12) = 7.163, *P* = 0.0202, respectively, Figure 2B].

To further evaluate mitochondrial morphology, we performed TEM on hippocampal sections. LPS exposure led to excessive mitochondrial fission, characterized by shorter and more punctate mitochondria in the LPS + vehicle group, in contrast to the elongated, tubular mitochondria observed in the Con + vehicle group (Figure 2C). Quantitative analysis revealed an increased number of mitochondria [*F* (1, 32) = 4.703, *P* = 0.0377, Figure 2D], and a reduced mitochondrial long diameter [*F* (1, 212) = 17.20, *P* < 0.0001, Figure 2E] in the LPS + vehicle group, both of which were significantly attenuated by Mdivi-1 treatment. No significant differences were found in the short diameter of mitochondria among the groups [*F* (1, 212) = 0.1231, *P* = 0.7260, Figure 2F].

Excessive mitochondrial fission has been associated with impaired mitochondrial function and reduced ATP production. To assess this, we measured ATP levels in hippocampal tissues and found that LPS significantly decreased ATP levels, indicating compromised energy metabolism. Notably, administration of Mdivi-1 effectively prevented LPS-induced ATP depletion [*F* (1, 20) = 12.90, *P* = 0.0018, Figure 2G], suggesting that inhibition of DRP1-mediated fission helps preserve mitochondrial energy output in the context of sepsis-associated neuroinflammation.

### 3.3 Mdivi-1 ameliorated LPS-induced DRP1 upregulation in PV interneurons in the mouse hippocampus

**FIGURE 3:**
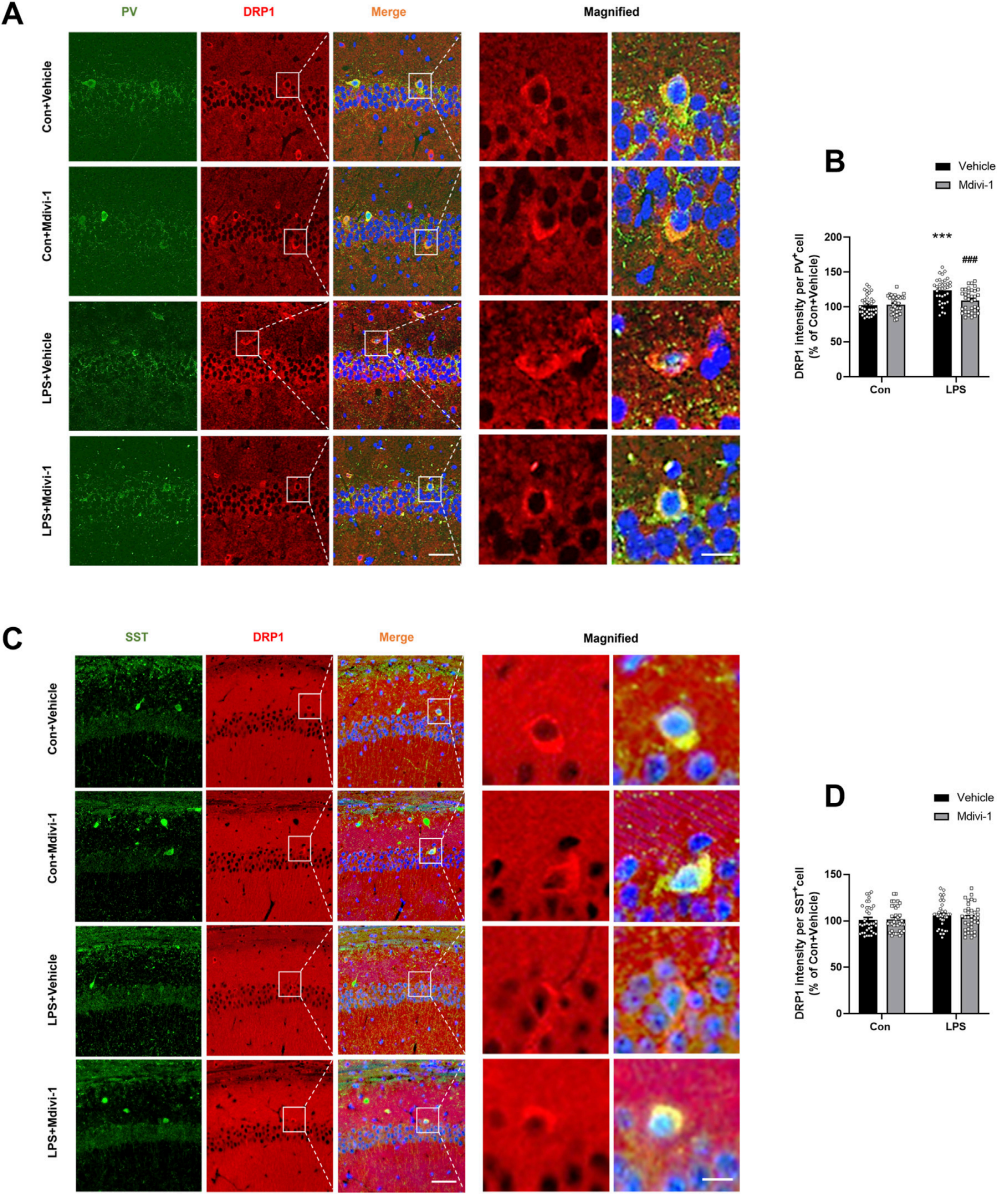
Mdivi-1 rescued LPS-induced DRP1 upregulation in PV interneurons in the mouse hippocampus. **(A)** Representative immunofluorescence images showing PV interneurons (green), DRP1 (red), DAPI (blue), and their colocalization in the hippocampal CA1 region. **(B)** Quantification of DRP1 fluorescence intensity in PV-positive cells across the four groups. **(C)** Representative immunofluorescence images showing SST interneurons (green), DRP1 (red), DAPI (blue), and their colocalization in the CA1 region. **(D)** Quantification of DRP1 fluorescence intensity in SST-positive cells across the four groups. Scale bar = 50 μm, the magnified figure scale bar = 10 μm. Data are presented as the mean ± SEM (n = 6 per group, two independent sections per mouse, three PV^+^ or SST^+^ cells randomly selected per section). ***P < 0.001 compared to the Con + vehicle group, ^###^ *P <* 0.001 compared to the LPS + vehicle group.

Previous studies have suggested that PV interneurons are particularly vulnerable to damage during systemic inflammation (Ji et al., 2020). Their high energy demands make them especially susceptible to mitochondrial dysfunction and energy deficits (Inan et al., 2016; Kontou et al., 2021). DRP1 has been implicated in neuronal damage, and inhibition of DRP1 can protect PV interneurons by reducing abnormal mitochondrial fission in various disease conditions, including status epilepticus (Kim and Kang, 2017). To assess DRP1 expression in specific neuronal subtypes and the effect of Mdivi-1, we performed double immunofluorescence staining for DRP1 with PV and SST, markers for parvalbumin (PV) and somatostatin (SST) GABAergic interneurons (Perez et al., 2019bib_perez_et_al_2019; Hudson et al., 2020), respectively, in the hippocampus and quantified DRP1 fluorescence intensity in these neuronal populations. Our results showed a significant increase in DRP1 expression within PV^+^ interneurons following LPS treatment. Notably, this LPS-induced elevation was significantly attenuated by Mdivi-1 pretreatment [*F* (1, 140) = 9.447, *P* = 0.0025, Figures 3A,B]. In contrast, there were no significant changes in DRP1 expression in SST^+^ neurons among the four groups [*F* (1, 140) = 0.2538, *P* = 0.6152, Figures 3C,D]. These findings suggest that LPS may selectively induce DRP1 upregulation in PV interneurons, and that Mdivi-1 effectively mitigates this effect.

### 3.4 Mdivi-1 attenuated LPS-induced downregulation of GAD67 and PV in the mouse hippocampus

**FIGURE 4:**
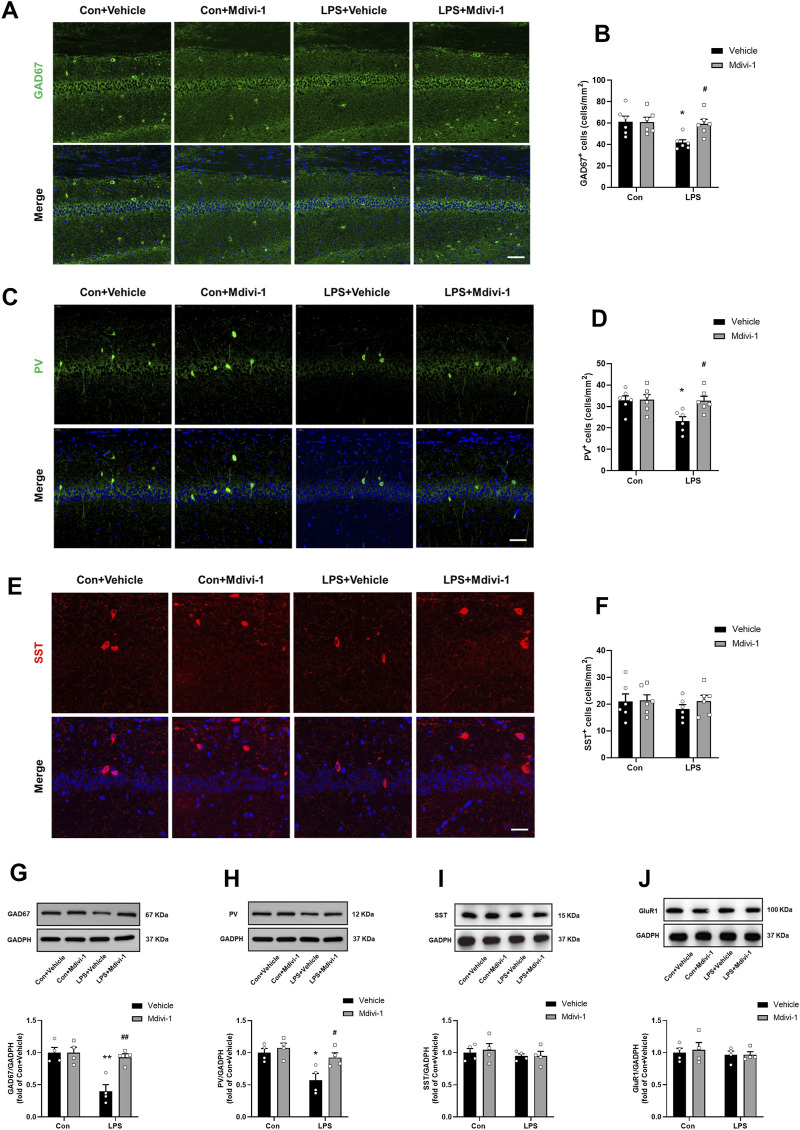
Mdivi-1 attenuated LPS-induced downregulation of GAD67 and PV in the mouse hippocampus. **(A)** Representative immunofluorescence images of GAD67-positive cells (green) in the hippocampal CA1 region. **(B)** Quantification of GAD67-positive cells in the CA1 region. **(C)** Representative immunofluorescence images of PV-positive cells (green) in the CA1 region. **(D)** Quantification of PV-positive cells in the CA1 region. **(E)** Representative immunofluorescence images of SST-positive cells (red) in the CA1 region. **(F)** Quantification of SST-positive cells in the CA1 region. DAPI (blue) was used for nuclear staining. Scale bar = 50 μm. Data are presented as the mean ± SEM (n = 6 per group). **(G–J)** Representative Western blotting and quantitative analysis of the protein levels of GAD67, PV, SST and GluR1 in the hippocampus. Data are presented as the mean ± SEM (n = 4 per group). *P < 0.05, **P < 0.01 compared to the Con + vehicle group, ^#^ *P <* 0.05, ^##^ *P <* 0.01 compared to the LPS + vehicle group.

To further evaluate the impact of excessive mitochondrial fission on GABAergic neuron populations in SAE progression, we performed immunostaining in the hippocampus using antibodies against GAD67 (a marker for GABAergic neurons), PV, and SST. Compared to the Con + vehicle group, the number of GAD67^+^ and PV^+^ staining cells were significantly decreased in the hippocampus of the LPS + vehicle group. Notably, Mdivi-1 treatment attenuated these reductions in both GAD67^+^ [*F* (1, 20) = 4.455, *P* = 0.0476, Figures 4A,B] and PV^+^ neurons [*F* (1,20) = 4.519, *P* = 0.0462, Figures 4C,D]. In contrast, the number of SST^+^ neurons remained unchanged across groups [*F* (1,20) = 0.3634, *P* = 0.5534, Figures 4E,F].

We next performed Western blotting to quantify the protein levels of GAD67, PV, and SST. In addition, we assessed GluR1 as a general marker of glutamatergic neurons (Gelders et al., 2018). Our results indicated that LPS did not significantly alter the expression of SST [*F* (1, 12) = 0.0070, *P* = 0.9346, Figure 4I] and GluR1 [*F* (1, 12) = 0.0017, *P* = 0.9680, Figure 4J]. Intriguingly, there was a significant reduction in the levels of GAD67 [*F* (1, 12) = 10.23, *P* = 0.0076, Figure 4G] and PV [*F* (1, 12) = 5.028, *P* = 0.0446, Figure 4H], suggesting that LPS may lead to dysregulation of PV interneurons. Notably, Mdivi-1 treatment attenuated the reductions in GAD67 and PV protein levels [*F* (1, 12) = 10.23, *P* = 0.0076; *F* (1, 12) = 5.028, *P* = 0.0446, respectively, Figures 4G,H].

These results suggest that LPS selectively downregulates PV interneurons in the hippocampus, and that Mdivi-1 treatment effectively attenuates this effect.

### 3.5 Mdivi-1 reversed LPS-induced alterations in neural oscillations in the hippocampus of mice

**FIGURE 5:**
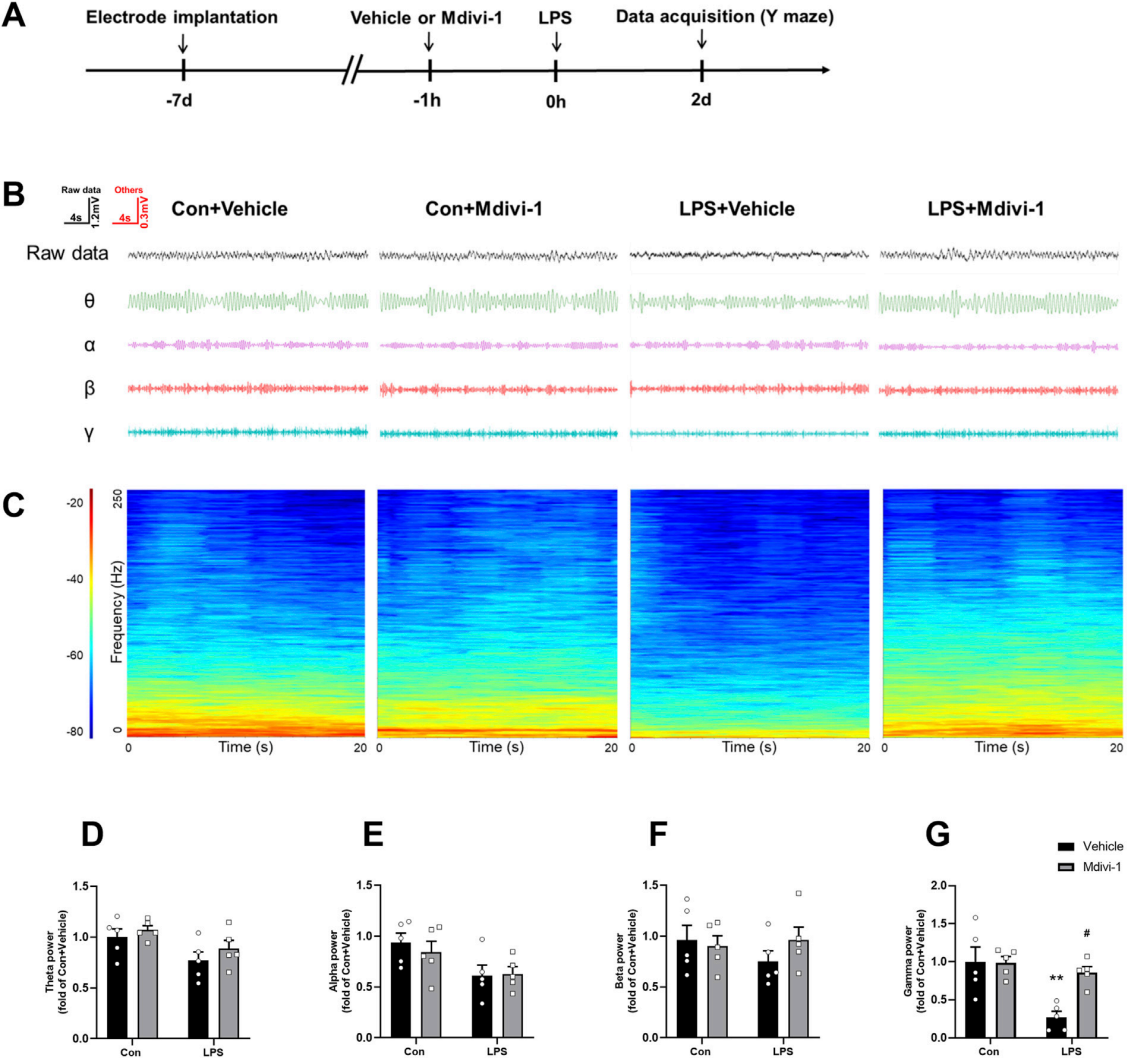
Mdivi-1 reversed LPS-induced decrease in gamma band power in the hippocampal CA1 region. **(A)** Schematic timeline of the electrophysiological experiment. **(B)** Representative traces of local field potentials (LFPs) and filtered signals for theta, alpha, beta, and gamma frequency bands in the hippocampus. **(C)** Time-frequency diagrams in the hippocampus. **(D–G)** Quantification of average theta, alpha, beta, and gamma band power in the hippocampus. Data are presented as the mean ± SEM (n = 5 per group). **P < 0.01 compared to the Con + vehicle group, ^#^ *P <* 0.05 compared to the LPS + vehicle group.

PV interneurons are known to play a crucial role in the synchronization of the hippocampal network (Ognjanovski et al., 2017), as well as in controlling principal cell activity and orchestrating neural oscillations in the hippocampal region (Khlaifia et al., 2022). Gamma oscillations, which are essential for cognitive processes, are driven by the reciprocal interaction between PV interneurons and principal pyramidal cells (Buzsaki and Draguhn, 2004). In contrast, SST interneurons are more closely linked to theta oscillations (Mikulovic et al., 2018). To further investigate the impact of Mdivi-1 on altered oscillatory activities, we performed LFP recordings in the hippocampal CA1 during the Y maze test. As shown in Figure 5, the LPS + vehicle group exhibited a significant reduction in gamma power compared to the Con + vehicle group, while theta power remained unchanged. Mdivi-1 treatment effectively reversed the gamma power deficit [theta: *F* (1, 16) = 0.0932, *P* = 0.7641; alpha: *F* (1, 16) = 0.3468, *P* = 0.5641; beta: *F* (1, 16) = 1.187, *P* = 0.2921; gamma: *F* (1, 16) = 6.552, *P* = 0.0210].

### 3.6 Mdivi-1 attenuated cognitive impairments induced by LPS in mice

**FIGURE 6:**
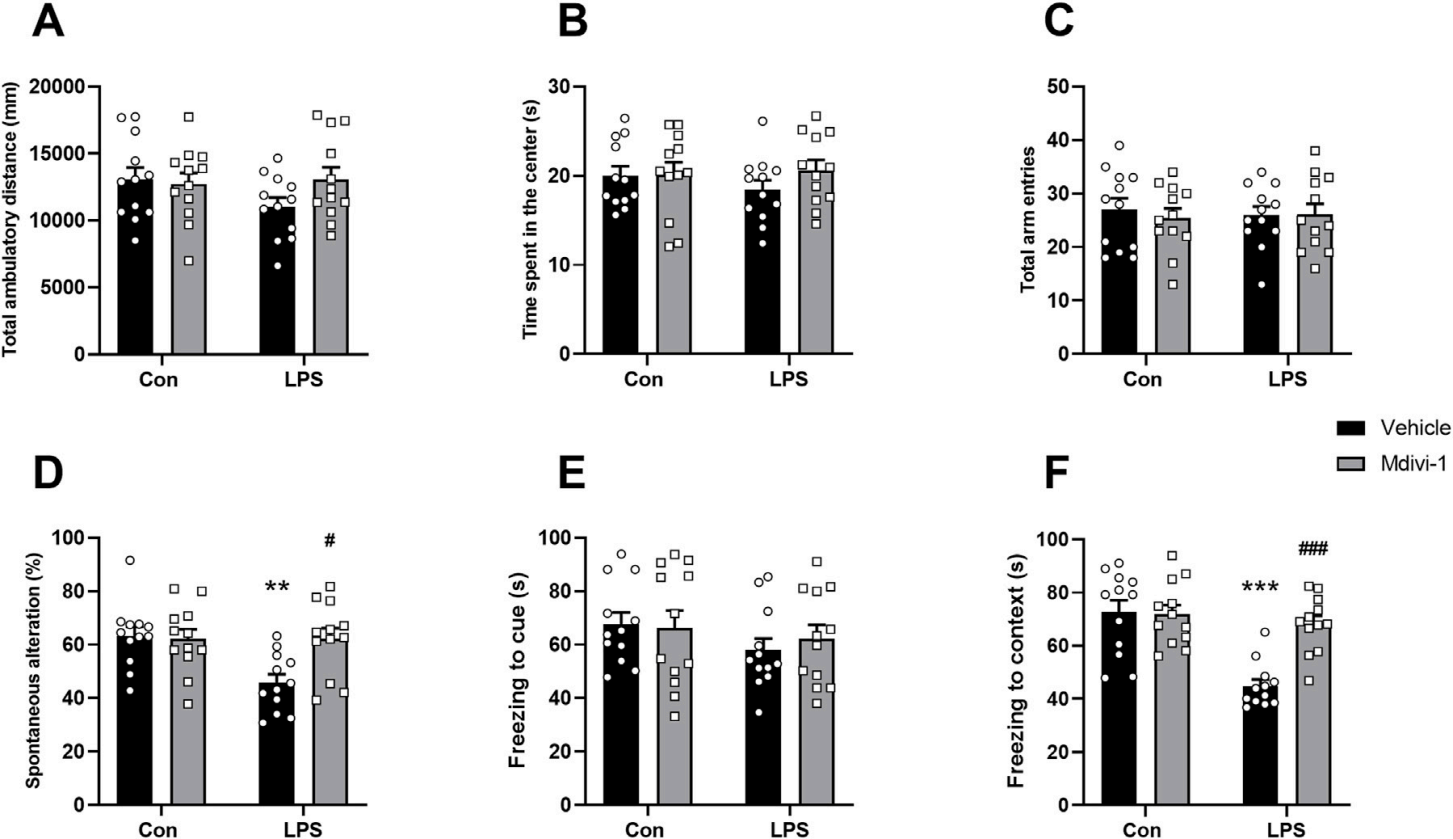
Mdivi-1 improved learning and memory impairments after LPS challenge in mice. **(A)** Total ambulatory distance and **(B)** time spent in the center during open field tests. **(C)** Total arm entries and **(D)** spontaneous alterations in Y-maze tests. **(E)** Freezing time to cue and **(F)** freezing time to context in fear conditioning tests. Data are presented as the mean ± SEM (n = 12 per group). **P < 0.05, ***P < 0.001 compared to the Con + vehicle group, ^#^ *P <* 0.05, ^###^ *P <* 0.001 compared to the LPS + vehicle group.

To assess the impact of LPS challenge and Mdivi-1 treatment on learning and memory functions, we subjected the mice to a series of behavioral tasks, as outlined in our previous research (Fu et al., 2019; Qiu et al., 2020). Locomotor activity and exploratory behavior were evaluated using the open field test to determine whether LPS or Mdivi-1 administration affected the mice’s performance. There were no significant differences in total ambulatory distance [*F* (1, 44) = 2.024, *P* = 0.1619, Figure 6A] or time spent in the center of the arena [*F* (1, 44) = 0.7581, *P* = 0.3886, Figure 6B] among the four groups. There was no significant difference in the total number of arm entries among the four groups [*F* (1, 44) = 0.2066, *P* = 0.6517, Figure 6C]. However, mice in the LPS + vehicle group displayed fewer spontaneous alternations compared to the Con + vehicle group, a deficit that was reversed by Mdivi-1 treatment [*F* (1, 44) = 6.074, *P* = 0.0177, Figure 6D]. The fear conditioning test was employed to evaluate hippocampus-related memory performance. There were no significant differences in the cue fear test among the four groups [*F* (1, 44) = 0.2976, *P* = 0.5882, Figure 6E], while the LPS + vehicle group exhibited a significant decrease in context freezing time compared to the Con + vehicle group. Notably, this reduction in freezing time was ameliorated by Mdivi-1 administration [*F* (1, 44) = 12.31, *P* = 0.0011, Figure 6F].

## 4 Discussion

**FIGURE 7:**
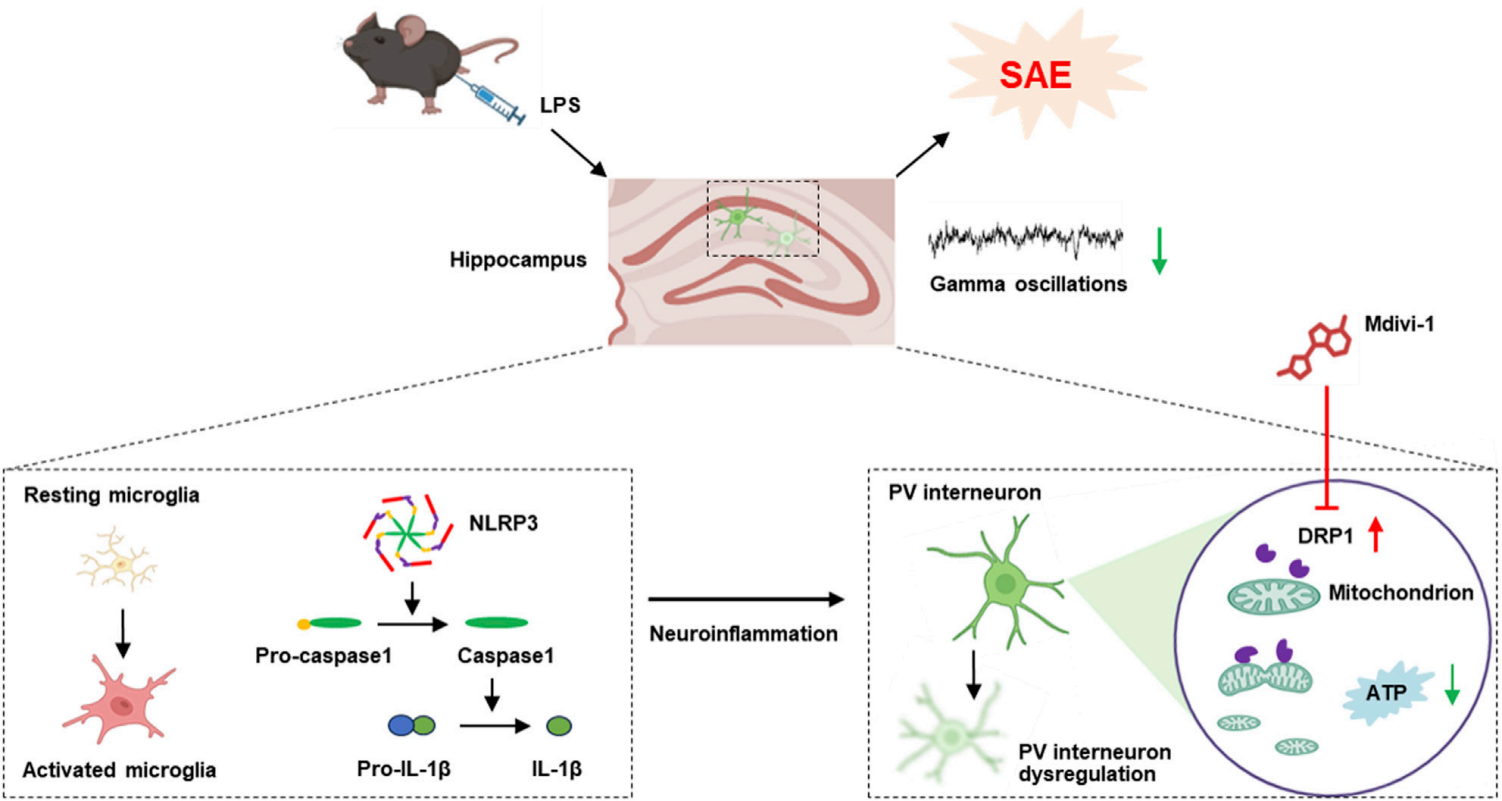
The schematic diagram illustrates that LPS activates microglia and NLRP3 inflammasome signaling, leading to excessive mitochondrial fission, reduced ATP production, and PV interneuron dysfunction. This dysregulation impaired gamma oscillations and contributed to cognitive deficits. Notably, Mdivi-1 treatment partially rescued PV interneuron function and improved cognition in this SAE model.

Our study demonstrates that LPS induces activation of microglial cells and NLRP3 inflammasome signaling, leading to excessive mitochondrial fission and reduced ATP production. This was accompanied by overexpression of the mitochondrial fission protein DRP1 specifically in PV interneurons and a downregulation of PV expression in the hippocampus. The dysregulation of PV interneurons contributed to impaired gamma oscillations, collectively resulting in cognitive impairments in mice. Notably, administration of the mitochondrial fission inhibitor Mdivi-1 at least partially rescued PV interneuron dysfunction and ameliorated cognitive deficits in this mouse model of SAE (Figure 7).

LPS is widely used to model the neuroinflammatory mechanisms underlying SAE. It is well established that LPS disrupts BBB integrity and triggers prolonged glial activation, leading to the accumulation of proinflammatory cytokines in the brain (Qin et al., 2007). The hippocampus, a region highly sensitive to inflammatory signaling due to its dense expression of cytokine receptors, is particularly vulnerable. In line with previous studies (Mao et al., 2021; Fu et al., 2019), our results showed that LPS induced NLRP3 inflammasome activation and microglial reactivity in the hippocampus, accompanied by impairments in spatial and associative memory, as shown by Y-maze and fear conditioning tests, supporting the validity of our SAE model.

Activation of the NLRP3 inflammasome promotes the release of IL-1β, which disrupts mitochondrial homeostasis by increasing oxidative stress, impairing mitochondrial membrane potential, and enhancing DRP1-mediated fission (Zhang et al., 2023; Miao et al., 2023). We recently showed that the NLRP3-GSDMD (Gasdermin D, an execution protein involved in inflammatory cell death) pathway can facilitate DRP1 activation and its translocation to mitochondria, leading to excessive mitochondrial fragmentation and energy deficits (Fu et al., 2024). Conversely, mitochondria can also promote NLRP3 activation by releasing intracellular signals such as mitochondrial DNA, ROS, and Ca^2+^, further amplifying neuroinflammation (Zhang Y.-F. et al., 2021; Zuo et al., 2020). In line with these findings, our results demonstrated that LPS challenge activated the NLRP3 inflammasome in the hippocampus, coinciding with upregulation of DRP1, downregulation of OPA1, increased mitochondrial number, reduced mitochondrial length, and decreased ATP production, supporting the presence of excessive mitochondrial fission and energy deficits. Notably, treatment with the DRP1 inhibitor Mdivi-1 attenuated these alterations, restoring mitochondrial integrity and ATP levels. These findings suggest that DRP1 inhibition may counteract NLRP3-driven neuroinflammation and mitochondrial dysfunction in SAE.

Among GABAergic interneurons, PV-expressing interneurons are particularly susceptible to inflammatory insults. PV is broadly expressed in brain regions including the hippocampus, and its dysfunction has been associated with various neurodegenerative and psychiatric disorders (Paterno et al., 2021). In this study, we observed a significant increase in DRP1 immunofluorescence intensity specifically in PV^+^ interneurons, but not in SST^+^ interneurons, suggesting that in our SAE model, DRP1 activation is largely restricted to PV interneurons. Correspondingly, we observed a marked reduction in the number of GAD67^+^ and PV^+^ neurons, as well as a decrease in GAD67 and PV protein expression following LPS administration, while SST levels remained unaffected. This selective vulnerability of PV interneurons aligns with prior LPS-induced SAE models (Ji et al., 2020; Mao et al., 2021). A plausible explanation for this vulnerability is the exceptionally high energy demand of PV interneurons and their reliance on precisely regulated mitochondrial dynamics (Ji et al., 2020; Inan et al., 2016). As DRP1 is a central mediator of mitochondrial fission, our cell-type-specific analysis supports the idea that mitochondrial dysfunction, particularly within PV neurons, plays a key role in SAE-associated cognitive impairment.

It is important to note that PV interneurons represent only a small fraction of the total hippocampal neuronal population. Therefore, the elevated DRP1 levels observed in our Western blot analyses may also reflect contributions from other neuronal or glial cell types not specifically examined in this study. Moreover, the effects of sepsis on neuronal subtypes can vary depending on the experimental model. For instance, repeated LPS exposure has been shown to upregulate GluR1 and GluR2 (He et al., 2024), whereas CLP (cecal ligation and puncture)-induced sepsis results in GluR1 downregulation (Qin et al., 2021). These discrepancies across studies may be attributed to differences in animal models (e.g., LPS vs CLP), LPS dosage, frequency of exposure, timing of tissue collection, and brain regions examined (Qin et al., 2021; He et al., 2024).

Targeting mitochondrial fission has been shown to protect PV interneurons in other disease models, such as status epilepticus (Kim and Kang, 2017). Mdivi-1 has been reported to inhibit DRP1 activation, preserve mitochondrial morphology, and reduce LPS-induced brain damage (Deng et al., 2018) and neuronal apoptosis in models of traumatic brain injury (Wu et al., 2016; Song et al., 2019). In the present study, Mdivi-1 effectively mitigated LPS-induced mitochondrial fission abnormalities and ATP depletion in the hippocampus and attenuated PV interneuron loss, as reflected by restored PV expression and increased number of PV^+^ cells. These findings suggest that the neuroprotective effects of Mdivi-1 in the SAE model may be mediated, at least in part, through the preservation of mitochondrial dynamics and PV interneuron integrity. As PV interneurons are essential for generating gamma oscillations in the hippocampus, which are critical for learning and memory (Cardin et al., 2009; Sohal et al., 2009). In our study, Mdivi-1 not only protected PV interneurons but also helped restore normal gamma oscillatory activity in the hippocampus following LPS challenge. While our *in vivo* gamma oscillation recordings indirectly suggest PV interneuron dysfunction, future studies should directly measure PV neuronal activity using approaches such as patch-clamp recordings in brain slices.

Notably, although the LPS model replicates key features of SAE-associated neuroinflammation, it does not fully mimic the polymicrobial infection and systemic disturbances seen in clinical sepsis. To enhance translational relevance, our previous work demonstrated that Mdivi-1 also alleviates mitochondrial fission abnormalities, synaptic damage, disrupted hippocampal oscillations, and cognitive deficits in a CLP model of sepsis (Fu et al., 2024). Consistent findings from other CLP studies further support that Mdivi-1 preserves mitochondrial integrity, reduces neuroinflammation, and improves cognitive function (Hong et al., 2025; Zhu et al., 2021). Together, these findings suggest that targeting mitochondrial fission with Mdivi-1 may be a broadly applicable therapeutic strategy for SAE.

This study has several limitations. First, we assessed GABAergic neuron subpopulations only at a single time point (24 h after LPS challenge); future studies should examine these alterations across multiple time points to capture dynamic changes. Second, while our immunostaining analysis of DRP1 focused on PV and SST interneurons, we cannot exclude potential effects of Mdivi-1 on other neuronal or glial cell types. Cell-type-specific approaches will be essential to identify the broader cellular targets of Mdivi-1 and further elucidate the role of DRP1 in SAE. Third, Mdivi-1 was administered only once without a dose-response study, and the long-term adverse effects of Mdivi-1 treatment have not been evaluated. Lastly, while the hippocampus is critical in cognitive impairments during sepsis, other brain regions may also be involved.

## 5 Conclusion

Our data suggest that Mdivi-1 administration effectively mitigates LPS-induced mitochondrial fission abnormalities, preserves PV interneuron integrity, and restores cognitive function in a mouse model of SAE, indicating that Mdivi-1 may represent a promising therapeutic approach for this condition.

## Data Availability

The original contributions presented in the study are included in the article/supplementary material, further inquiries can be directed to the corresponding authors.
